# Endoscopic ultrasound with tissue acquisition of lymph nodes in patients with potentially resectable intrahepatic cholangiocarcinoma

**DOI:** 10.1055/a-2366-2592

**Published:** 2024-08-23

**Authors:** David M. de Jong, Sanne van de Vondervoort, Roy S. Dwarkasing, Maarten G.J. Thomeer, Michael Doukas, Rogier P. Voermans, Robert C. Verdonk, Wojciech G. Polak, Jeroen de Jonge, Marco J. Bruno, Lydi M.J.W. Van Driel, Bas Groot Koerkamp

**Affiliations:** 1 Department of Gastroenterology and Hepatology, Erasmus MC University Medical Center, Rotterdam, Netherlands; 2 Department of Radiology and Nuclear Medicine, Erasmus MC University Medical Center, Rotterdam, Netherlands; 3 Department of Pathology, Erasmus MC University Medical Center, Rotterdam, Netherlands; 4 Department of Gastroenterology and Hepatology, Amsterdam University Medical Center, Amsterdam, Netherlands; 5 571165 Amsterdam Gastroenterology Endocrinology Metabolism, Amsterdam, Netherlands; 6 571143 Cancer Treatment and Quality of Life, Cancer Centre Amsterdam, Amsterdam, Netherlands; 7 6028 Department of Gastroenterology and Hepatology, Sint Antonius Hospital, Nieuwegein, Netherlands; 8 Transplantation Institute, Department of Surgery, Erasmus MC University Medical Center, Rotterdam, Netherlands; 9 Cancer Institute, Department of Surgery, Erasmus MC University Medical Center, Rotterdam, Netherlands

**Keywords:** Endoscopic ultrasonography, Biliary tract, Tissue diagnosis, Fine-needle aspiration/biopsy, GI surgery

## Abstract

**Background and study aims** Lymph node (LN) involvement is a poor prognostic factor for patients with intrahepatic cholangiocarcinoma (iCCA). The aim of this study was to evaluate the yield and impact on clinical decision making of endoscopic ultrasound with tissue acquisition (EUS-TA) of LNs in patients with potentially resectable iCCA.

**Patients and methods** In this multicenter cohort study, patients with potentially resectable iCCA and preoperative EUS between 2010 and 2020 were retrospectively included. The impact of EUS-TA was defined as the percentage of patients who did not undergo surgical exploration due to pathologically confirmed positive LNs found with EUS-TA.

**Results** A total of 56 patients underwent EUS, with 91% of patients to target suspicious LNs on imaging. EUS-TA of LNs confirmed malignancy in 21 LNs among 19 patients (34%). In 17 patients (30%), surgical exploration was withheld due to nodal involvement. Finally, 24 patients (43%) underwent surgical exploration among whom positive regional LNs were identified in six patients (25%).

**Conclusions** In patients with potentially resectable iCCA and suspicious LNs on cross-sectional imaging, EUS-TA confirmed LN involvement in 30% of patients. Surgical exploration was withheld mostly because of extraregional LN involvement and regional LN involvement in patients with high surgical risk.

## Introduction

Intrahepatic cholangiocarcinoma (iCCA) is a rare malignancy originating from the intrahepatic biliary tree proximal to the second-order bile ducts. A complete surgical resection is performed in about 20% of patients with iCCA, with a 5-year survival rate of 30.4% 1 . Most patients present with locally advanced disease or distant metastasis 2 .

The presence of positive lymph nodes (LNs) is a poor prognostic factor for iCCA 3 . In the 7 ^th^ edition of the American Joint Committee on Cancer (AJCC) staging system for iCCA, N status was determined by the location of a positive LN; N1 for regional LN and N2 for extraregional LN (including aortocaval and celiac) 4 . In the 8 ^th^ edition, N stage only reflects the number of regional positive LNs, whereas extraregional positive LNs are classified with all other distant metastases (i.e., stage IV). Positive extraregional LNs are a contraindication to surgical resection in most patients 5 .

Approximately 40% of patients who undergo iCCA resection are found to have positive regional LNs 6 . These patients also have a poor median overall survival (OS)after surgical resection of only 18 months versus 45 months if LNs are negative 1 . The 5-year cancer-specific survival for patients with LN metastases is 13.1% compared with 44.9% for no LN metastasis 7 . This poor OS may not justify a major liver resection, especially in patients with high surgical risk 8 . Endoscopic ultrasound with tissue acquisition (EUS-TA) could confirm positive regional LN in these patients and avoid surgical exploration.

Cross-sectional imaging with computed tomography (CT) or magnetic resonance imaging (MRI) has limited accuracy for detection of positive LNs 9 . In other gastrointestinal malignancies such as esophageal cancer, EUS-TA is often performed to evaluate LN status 10 11 12 . Several studies have reported promising results for use of EUS in the preoperative setting for cholangiocarcinoma 10 11 13 14 . The recently published clinical practice guidelines on management of iCCA by the European Association for the Study of the Liver (EASL) and International Liver Cancer Association (ILCA) recommend that patients with apparent resectable iCCA undergo LN sampling by EUS-TA during staging evaluation, if a positive result would alter management 15 . However, data on the use of preoperative LN staging in the setting of iCCA are limited to one study and the impact on clinical decision making is still unclear 11 . Therefore, our objective was to assess the yield and its impact on clinical decision making of EUS-TA of LN in patients with potentially resectable iCCA.

## Patients and methods

### Study population

We conducted a retrospective, multicenter cohort study at three Dutch tertiary referral centers. All consecutive patients with suspected potentially resectable iCCA who underwent an EUS preoperatively and were discussed at a multidisciplinary meeting between January 2010 and June 2020 were eligible for inclusion. Patients with advanced iCCA (i.e., unresectable or stage IV) on imaging or with neoadjuvant treatment prior to EUS were excluded. Patients were identified by searching endoscopy report databases and electronic medical records. The study was conducted in accordance with the guidelines of the Helsinki Declaration and approved by the local ethics committees (MEC-2020–0963). Need for informed consent was waived due to the retrospective nature of the study.

### Regional and extraregional LN locations

**Fig. 1:**
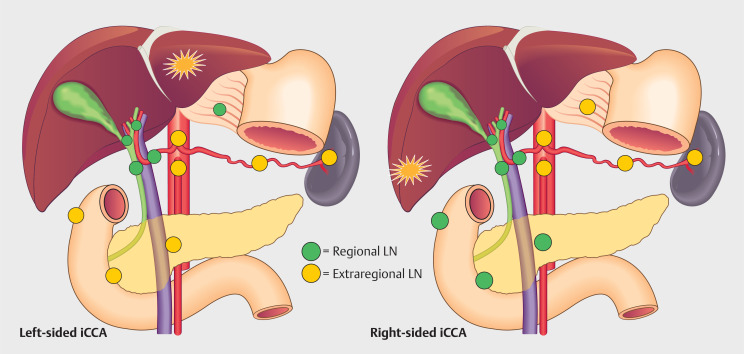
Figure showcasing the locations of regional and extraregional LN for both left- and right-sided iCCA.

LN locations were defined according to the 8 ^th^ AJCC edition ( Table 1 ) 4 16 . Regional and extraregional LNs were defined differently for iCCA located in the left or right hemi-liver ( Fig. 1 ). LN locations not covered by the AJCC classification were noted separately and considered extraregional if located distally from the furthest possible regional LN for left- or right-sided iCCA.

**Table:** **Table 1** AJCC staging system classification regarding LN status for iCCA.

	**7th edition**		**8th edition**	
	N1	M1	N1	M1
Left liver iCCA (segment 2–4)	≥1 LNM in the regional LNs (hilar, CD, CBD, HA, PV, IP or GH LNs)	Distant metastasis (includes LNM in the CO, PA or PC LNs)	≥1 LNM in the regional LNs (hilar, CD, CBD, HA, PV, IP or GH LNs)	Distant metastasis (includes LNM in the CO, PA or PC LNs)
Right liver iCCA (segment 5–8)	≥1 LNM in the regional LNs (hilar, CD, CBD, HA, PV, PD or PP LNs)	Distant metastasis (includes LNM in the CO, PA or PC LNs)	≥1 LNM in the regional LNs (hilar, CD, CBD, HA, PV, PD or PP LNs)	Distant metastasis (includes LNM in the CO, PA or PC LNs)
CBD, common bile duct; CD, cystic duct; CO, celiac; GH, gastrohepatic; HA, hepatic artery; IP, inferior phrenic; LN, lymph node; LNM, lymph node metastasis; Nx, regional lymph nodes cannot be assessed; N0, no regional lymph node metastasis; PA, periaortic; PC, pericaval; PD, periduodenal; PP, peripancreatic; PV, portal vein.				

### EUS procedure and work-up for surgery

The indication for EUS was LN assessment due to regional or extraregional lymphadenopathy identified on imaging or assessment of the primary iCCA lesion or additional lesions that may reflect multifocal disease. A systematic and comprehensive LN assessment was not always performed. The EUS procedure could be performed at one of the three study sites or at one of the referral hospitals. At the three study sites all procedures were performed by an experienced endosonographer (>1000 lifetime procedures). Moreover, number and location of LNs was not systematically described in the reports. The procedures were performed using a linear ultrasound endoscope (Olympus GF-UCT-160 or GF-UCT-180 and Pentax EG-3870 UTK, EG-3270 UK or EG38-J10 UT). Suspicious LNs were defined as having one or more of the following characteristics: short axis diameter >5 mm, hypoechoic, round shape, and clear demarcation. EUS-TA was routinely performed in suspicious LNs, using 19-, 20-, 22- or 25-gauge fine-needle aspiration or fine-needle biopsy needles from Cook Medical. The pathologists at all three study sites, with a subspecialization in hepato-biliary neoplasms, categorized the tissue obtained by EUS-TA as positive (malignancy), negative (no malignancy), or nondiagnostic (not enough cells to make a diagnosis).

Cross-sectional imaging was routinely performed before EUS by CT and/or MRI with or without magnetic resonance cholangiopancreatography. Radiologists defined lymphadenopathy as suspicious LN based on location, heterogeneity, and size criteria (>1 cm). Often the number and specific location of the lymphadenopathy was not described in the radiological reports. Patients with biopsy-confirmed positive extraregional LNs were not routinely considered for surgical resection. The decision to proceed with surgical exploration for patients with biopsy-confirmed positive LNs was made by multidisciplinary discussion and shared decision making with the patient. Neoadjuvant chemotherapy was not routinely considered with biopsy-confirmed positive LNs.

### Outcome definition

The primary study outcome was the impact of EUS-TA on clinical decision making, which was defined as the number of patients for whom surgical exploration was withheld due to pathological confirmation of positive regional and extraregional LNs with EUS-TA divided by the total number of patients who underwent EUS.

### Data collection

Data were collected on patient and disease demographics (age, sex, primary sclerosing cholangitis (PSC) diagnosis, performance status according to the World Health Organization (WHO) and American Society of Anesthesiologists Physical Status Classification System [ASA]). All data on LN described at imaging, EUS, and surgical procedures were collected. The following information about the EUS procedure was collected: center where EUS was performed (tertiary referral center or referring hospital), total number of EUS procedures per patient, presence of drainage prior to EUS (via endoscopic retrograde cholangiography stent), locations of suspicious LNs, proportion of LN biopsy per location, and EUS-TA-related complications.

### Statistical analysis

Descriptive statistics were used. Categorical and dichotomous variables were described using frequencies and proportions, whereas continuous data were described using medians with interquartile ranges for non-normally distributed variables and means with standard deviations for normally distributed variables.

## Results

### Baseline characteristics

A total of 56 patients with presumed resectable iCCA who underwent EUS preoperatively were identified and included. Lymphadenopathy was identified in 91% of patients at cross-sectional imaging before the EUS was performed. Baseline characteristics are presented in Table 2 .

**Table:** **Table 2** Baseline characteristics of study population.

	All patients with resectable iCCA with preoperative EUS performed (n=56)
Age at diagnosis, median (IQR), years	64 (IQR: 56–72)
Female sex – n (%)	32 (57%)
PSC – n (%)	7 (13%)
Cirrhosis – n (%)	5 (9%)
ASA – n (%)	
1	11 (19%)
2	35 (63%)
3	10 (18%)
WHO – n (%)	
0	31 (55%)
1	20 (36%)
2–3	5 (9%)
Cross-sectional Imaging – n (%)	
CT only	21 (38%)
MRI/MRCP	2 (4%)
Both	33 (59%)
**Based on cross-sectional imaging**	
Tumor location – n (%)	
Left-sided	21 (37%)
Right-sided	35 (63%)
Long axis primary tumor size, median (IQR), mm	60 (IQR: 45–82)
Vascular involvement – n (%)	29 (52%)
Visceral peritoneum involvement – n (%)	11 (19%)
Direct invasion extrahepatic bile duct(s) – n (%)	15 (27%)
Number of liver tumors on imaging – n (%)	
1	51 (91%)
2	5 (9%)
AJCC (8 ^th^ edition) cT stage	
cT1a	6 (11%)
cT1b	7 (13%)
cT2	17 (30%)
cT3a	26 (46%)
Lymphadenopathy described on cross-sectional imaging – n (%)	51 (91%)
Number of EUS procedures per patient – n (%)	
One	48 (86%)
Two	7 (13%)
Three	1 (2%)
AJCC, American Joint Committee on Cancer; ASA, American Society of Anesthesiologists; CT, computed tomography; EUS, endoscopic ultrasound; iCCA, intrahepatic cholangiocarcinoma; IQR, interquartile range; MRCP, magnetic resonance cholangiopancreatography; MRI, magnetic resonance imaging; PSC, primary sclerosing cholangitis; WHO, World Health Organization.	

### EUS procedures

A total of 65 EUS procedures were performed in 56 patients ( Table 3 ). In eight patients (14%) more than one EUS was performed because the former procedure was inadequate or repeat EUS-TA was indicated. Of the 56 patients, one or more extraregional LNs were visualized in 34 patients (61%), 12 patients (21%) in whom only regional LNs were identified, and 10 patients (18%) in whom no LNs were identified or described during EUS. In 15 (44%) of the 34 patients with extraregional LNs at EUS, one or more regional LNs also were described. A total of 71 LNs were described in 46 patients (82%). In 55 of 71 LNs (78%), TA was successfully performed, in four LNs (6%) TA was not safely possible, and in 12 (17%) no TA was performed, but specific reasons were unclear. The outcomes per LN are described in Table 3 . In five patients (9%) TA of the primary liver tumor was performed, which showed malignancy in 80%. One EUS procedure was terminated early, but after the indicated TA, due to oxygen desaturation of the patient. There were no other complications associated with the EUS.

**Table:** **Table 3** Characteristics of 65 EUS procedures.

Variable	Total EUS procedures (n=65)
Location of EUS – n (%)	
Tertiary referral center	46 (71%)
Referring hospital	19 (29%)
ERCP stent prior to EUS – n (%)	5 (8%)
LN described at EUS – n (%)	51 (78%)
EUS-TA of liver tumor – n (%)	5 (8%)
FNA	2 (40%)
Positive	1 (50%)
FNB	3 (60%)
Positive	3 (100%)
Complication – n (%)	1 (1.5%)
EUS, endoscopic ultrasound; ERCP, endoscopic retrograde cholangiopancreatography; EUS-TA, EUS-guided tissue acquisition; FNA, fine-needle aspiration; FNB, fine-needle biopsy; LN, lymph node.	

### Yield of EUS-TA

**Fig. 2:**
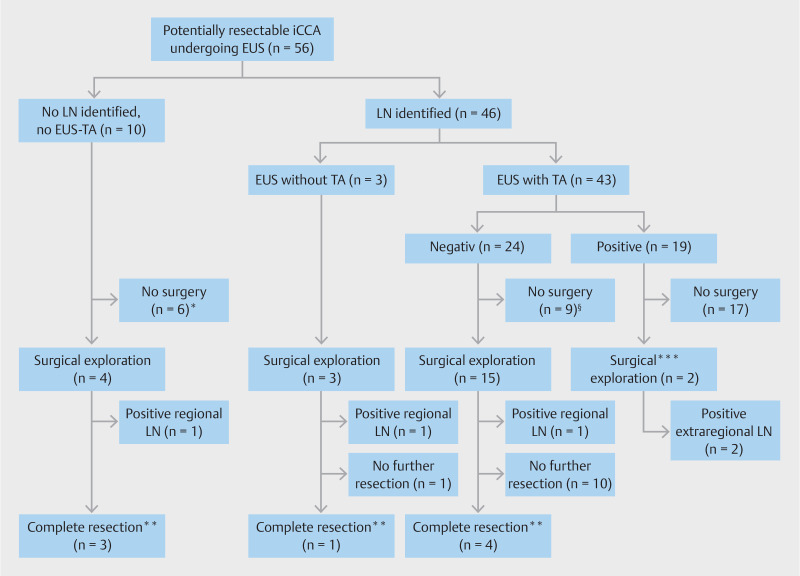
Flowchart of patients included in this study, according to EUS findings. *Surgery refusal (n = 3), disease progression during work-up showing unresectable disease (n = 2) and distant thoracic metastasis identified during work-up by percutaneous biopsy (n = 1). §Surgery refusal (n = 3), disease progression during work-up showing unresectable disease (n = 3), liver metastasis identified during work-up (n = 1), distant abdominal malignant LN identified by percutaneous biopsy (n = 1) and suspected Fasciola Hepatica diagnosis (n = 1). **Without positive regional LN at pathology assessment. ***Positive regional LN located next to the left liver lobe in which the iCCA was located (n = 1) and positive extraregional LN peri-pancreatic in a left-sided iCCA (n = 1)

EUS-TA showed malignancy in 21 of 55 LNs (38%), no malignancy in 30 LNs (55%), and was nondiagnostic in four LNs (7%). Positive extraregional LNs were identified by EUS-TA in 15 of 56 patients (27%) and positive regional LNs in five of 56 patients (9%). Overall, in 19 of 56 patients (34%) EUS-TA identified malignancy in LN because one patient had both ( Table 4 ). Fig. 2 shows the clinical course of all patients. After EUS, 17 patients (30%) were precluded from surgical exploration due to positive EUS-TA ( **Table S1** ) and 15 patients (27%) were precluded from surgery for various other reasons, as shown in Fig. 2 .

**Table:** **Table 4** Characteristics of all identified LNs based on EUS characteristics.

Described LN on EUS	#	No EUS-TA		Successful EUS-TA			Pathology results		
		Not possible	Not performed	FNA	FNB	Both	Positive	Negative	Non diagnostic*
Regional									
Suspicious	26	1	8	14	3		5	11	1
Not suspicious	3		2	1				1	
Extraregional									
Suspicious	36	2	2	31	3	2	16	17	3
Not suspicious	2	1	1						
EUS, endoscopic ultrasound; EUS-TA, EUS-guided tissue acquisition; FNA, fine-needle aspiration; FNB, fine-needle biopsy; LN, lymph node. *All nondiagnostic pathology results were from FNA.									

Finally, 24 patients (43%) underwent surgical exploration of whom two patients had prior EUS-TA-proven positive LN. These two patients with preoperatively confirmed positive LNs (one regional and one extraregional) underwent surgical exploration, but in both patients, multiple extraregional positive LN were found and the resection was thus cancelled.

Among the remaining 22 patients who underwent surgical exploration, in 14 patients (63.6%) no complete resection was performed. This was due to occult metastasis in the liver or peritoneum (n=5), locally advanced (i.e. unresectable) disease (n=4), in whom one patient had severely enlarged suspicious regional LNs behind the portal vein on palpation but biopsy was not safely possible and patient preference to refrain from further surgical treatment after separate staging surgery (n=2). In three patients, positive regional LNs were identified during the procedure. Further resection was not performed in these patients as all three had high surgical risk due to comorbidities.

Finally, in eight of the 22 patients (36%) a complete resection was performed, without nodal involvement. In one patient with a resection, final pathology showed no malignancy but sarcoidosis.

### Positive LN identified at surgery

At explorative surgery, positive LNs that were not identified with EUS-TA were found in three patients (13.6%). In the first patient, at explorative surgery with frozen section analysis, a positive hilar LN was identified. This LN was identified at CT and EUS, but was described as not suspicious. The second patient had a positive LN at frozen section analysis located at the common hepatic artery, but this LN was not identified on cross-sectional imaging and EUS. This patient underwent EUS-TA of regional and extraregional LNs at other locations, showing benign disease in both. The third patient had multiple positive LNs at the hepatic artery and portal vein in the surgical resection specimens, which were not identified at cross-sectional imaging and EUS.

## Discussion

EUS-TA confirmed positive LNs in 34% of patients with potentially resectable iCCA and suspicious LNs on cross-sectional imaging. Positive extraregional LNs were found in 27% of patients. Because of positive LNs confirmed with EUS-TA, surgical exploration was withheld in 30% of patients. These patients could proceed with more appropriate palliative systemic treatment and avoid staging laparoscopy or surgical exploration.

In a recent retrospective study by Malikowski et al., LNs were visualized during EUS in 20 of 24 iCCA patients (83%) 11 . EUS-TA identified positive LNs in six of 49 (12%) LNs biopsied, withholding surgical exploration in four of 24 patients (17%). The authors performed EUS-TA in all identified LNs after systematic mapping. However, this study did not distinguish between regional and extraregional LNs, or the location of the primary iCCA 11 . Patients with positive extraregional LNs have stage IV disease and are unlikely to benefit from surgical resection of iCCA. This concerned 79% of patients in the present study. Patients with positive regional LNs, however, can expect an OS after surgical resection that is considerably worse than with negative regional LNs, but still better than a median OS of 17 months with palliative treatment 1 17 . An individual approach with shared decision making is required to consider both surgical risk and potential oncological benefit of surgical resection.

In patients without preoperative confirmation of positive LNs, positive regional LNs were identified during surgical staging in 13.6% of patients. This rate is not directly comparable with the study of Malikowski et al. due to the abovementioned differences in study design. Malikowski et al. report the number of missed LNs for all cholangiocarcinoma subtypes 11 . Of the 130 patients without positive LNs by EUS-TA, 80 (62%) proceeded to staging laparotomy, with identification of positive LNs in four patients (5%). False-negative LN assessment can be explained by inadequate EUS, false-negative TA, or progression of disease in the time between EUS and the staging procedure. A recent study found that positive extraregional LNs precluding complete resection were identified in 11% of patients at surgical staging when EUS was not performed 18 .

This study is the first study on the yield and impact of preoperative EUS in patients with potentially resectable iCCA, distinguishing extraregional from regional LNs. The study population included all potentially resectable iCCAs on cross-sectional imaging with suspicious LN, rather than only patients with resected iCCA. Therefore, the impact of preoperative EUS-TA on clinical decision making could be assessed. This study also has several limitations. First, the retrospective nature of the study limited the data availability on LN location on cross-sectional imaging, EUS, and during surgery. Therefore, diagnostic test characteristics for each specific LN location could not be compared across imaging modalities. Use of four different needle sizes could potentially have a confounding effect, but we were unable to highlight the relative results regarding diagnostic accuracy. Second, not all consecutive patients with potentially resectable iCCA were included at the treatment centers, but only the patients in whom an EUS was performed. The indication for EUS was mostly lymphadenopathy on cross-sectional imaging. Therefore, the yield and effect on clinical decision making has likely been overestimated, compared with an approach in which all patients would undergo EUS-TA, regardless of lymphadenopathy seen on cross-sectional imaging. We were unable to include patients with presumed resectable iCCA who did not undergo EUS. Third, at the time of the EUS procedures included in this study, not all LNs were systematically assessed during EUS, making comparison with available results in the literature challenging 11 .

## Conclusions

In conclusion, the yield of EUS-TA to confirm positive LN in patients with potentially resectable iCCA was 38% and surgical exploration was withheld based on this result in 30% of patients. Prospective studies should be performed using a systematic approach with accurate description of LN location for each imaging modality. Such a clinical trial has started in the Netherlands, based on the results of this study (ClinicalTrials.gov NCT05678218).

### Data availability statement

The data that support the findings of this study are available on request from the corresponding author B. Groot Koerkamp.

## Supplementary Material

Supplementary Material Zusatzmaterial
