# Supplementary material for: Endoscopic ultrasound with tissue acquisition of lymph nodes in patients with potentially resectable intrahepatic cholangiocarcinoma
Source: Endosc Int Open. 2024 Aug 23;12(8):E998–E1005. doi: 10.1055/a-2366-2592 (PMC11343620; doi:10.1055/a-2366-2592)
Supplement: Supplementary file 1 — Supplementary Material [file 10-1055-a-2366-2592_23693235.pdf.pdf]

## Supplementary material

**Table S1** Patient characteristics for which EUS-TA precluded surgical exploration.

| Patient | Age, sex | ASA | WHO PS | cT stage | Longest tumor axis in mm | Number of tumors | Location in liver | Resection intention | EUS-TA location | (Extra) regional | Treatment afterward |
|---|---|---|---|---|---|---|---|---|---|---|---|
| 1 | 76, F | 2 | 0 | 1A | 20 | 1 | Left | S2/3 resection | Between left gastric and hepatic artery | ER | pCTx |
| 2 | 53, F | 1 | 0 | 1B | 50 | 1 | Left | Hemi | Celiac trunk + gastrohepatic | ER | pCTx/RTx |
| 3 | 79, M | 2 | 1 | 1B | 53 | 1 | Right | Hemi | Periaortic | ER | pCTx |
| 4 | 60, F | 2 | 0 | 1B | 80 | 1 | Right | Hemi | Left gastric artery | ER | pCTx |
| 5 | 34, F | 2 | 0 | 2 | 33 | 1 | Left | Hemi | Celiac trunk | ER | pCTx |
| 6 | 69, F | 2 | 0 | 2 | 37 | 2 | Left | Ext Hemi | Celiac trunk | ER | pCTx |
| 7 | 50, F | 1 | 0 | 2 | 118 | 1 | Right | Hemi | Pericaval | ER | BSC |
| 8 | 67, F | 2 | 0 | 3 | 67 | 1 | Right | Hemi | Periaortic | ER | pCTx |
| 9 | 76, F | 3 | 1 | 3 | 37 | 1 | Right | Hemi | Gastroduodenal artery | ER | pCTx |
| 10 | 61, M | 1 | 0 | 3 | 45 | 1 | Left | Hemi | Celiac trunk | ER | pCTx |
| 11 | 75, F | 2 | 1 | 3 | 53 | 2 | Right | Ext Hemi | Aortocaval | ER | BSC |
| 12 | 36, M | 2 | 1 | 3 | 81 | 1 | Right | Ext Hemi | Esophagus | ER | BSC |
| 13 | 73, M | 1 | 0 | 3 | 136 | 1 | Right | Ext Hemi | Celiac trunk | ER | BSC |

## Supplementary material

| | | | | | | | | | | | |
|---|---|---|---|---|---|---|---|---|---|---|---|
| 14 | 61, F | 2 | 1 | 1B | 52 | 1 | Right | Hemi | Portal vein + periduodenal | R | pCTx |
| 15 | 78, M | 2 | 1 | 2 | 41 | 1 | Right | Hemi | Hilar | R | BSC |
| 16 | 72, M | 2 | 0 | 3 | 85 | 1 | Right | Hemi | Hilar | R | pCTx |
| 17 | 70, M | 2 | 0 | 3 | 74 | 2 | Right | Ext hemi | Portal vein | R | pCTx |

BSC, best supportive care; Ext hemi, extended hemi-hepatectomy; Hemi, hemi-hepatectomy; NR, extraregional; pCTx, palliative chemotherapy; R, regional.
